# Expert recommendations on the management of major and minor toxicities associated with the use of lorlatinib in Latin America

**DOI:** 10.3389/fonc.2026.1740218

**Published:** 2026-10-26

**Authors:** Luis Corrales, Aknar Freire de Carvalho Calabrich, Aldo Agustin Perfetti, Luis Alberto Mas López, Javier Quilodran Peredo, Lizbett Vanessa Garcia Montes, Andrés Felipe Cardona Zorrilla, Sergio Andrés Mejía Espinosa, Maritza Ramos-Pérez, Carmen Sofía Pupareli, Gulherme Harada

**Affiliations:** 1 Centro de Investigación y Manejo del Cáncer (CIMCA), San José, Costa Rica; 2 Oncology Service, Clinica AMO, Salvador, Brazil; 3 Centro de Educación Médica de Investigaciones Clínicas (CEMIC), Buenos Aires, Argentina; 4 Oncosalud-AUNA, Lima, Peru; 5 Oncology Department, Clínica Alemana, Santiago de Chile, Chile; 6 Medical Oncology Department, Oncare, Ciudad de Mexico, Mexico; 7 Centro de Tratamiento e Investigación sobre Cáncer Luis Carlos Sarmiento Angulo (CTIC), Bogota, Colombia; 8 Clinical Oncology Unit, Centro de excelencia tumores torácicos, Clínica las Américas-AUNA, Medellín, Colombia; 9 Medical Oncology Department, Instituto Nacional de Cancerología, Ciudad de México, Mexico; 10 Medical Oncology Service, Instituto Alexander Fleming, Buenos Aires, Argentina; 11 Oncology Department, Hospital Sírio-Libanês, Sao Paulo, Brazil

**Keywords:** Latin America, lorlatinib, management, monitoring, toxicity

## Abstract

Lorlatinib is a third-generation anaplastic lymphoma kinase (ALK)/ROS1 tyrosine kinase inhibitor (TKI) that has been shown to have high efficacy in the first line setting for treatment of advanced ALK-rearranged non-small cell lung cancer (NSCLC). However, some adverse events have been described that require proper follow-up and management to obtain the maximum benefit from treatment and minimize risk to the patient. These include dyslipidemia and weight gain, conditions that in Latin America have a high prevalence in the general population, which coupled with limitations in timely access to specialized care, could influence both manifestation and management. The objective of this consensus is to provide recommendations tailored to our real-world setting that will optimize the monitoring and early management of toxicities in patients treated with lorlatinib in Latin America.

## 1 Introduction

The treatment of advanced non-small cell lung cancer (NSCLC) with an anaplastic lymphoma kinase (ALK+) gene rearrangement has undergone substantial evolution since the development of crizotinib, a first-generation ALK tyrosine kinase inhibitor (TKI). Presently, three generations of ALK-TKIs are available, and each new generation has demonstrated increased potency, enhanced central nervous system penetration, and improved ability to overcome ALK-resistant mutations (1, 2). Given the limited access to certain tests, sequencing could be a good option in many places in Latin America. We know that 30% of patients are still lost from first-line to second-line.

Lorlatinib is a highly potent, third-generation ALK/ROS1 tyrosine kinase inhibitor (TKI) with high central nervous system penetration and the ability to overcome most known resistance mutations that may develop during treatment with first- and second-generation ALK TKIs (3, 4).

The safety profile of lorlatinib is distinct and includes hyperlipidemia, edema, weight gain, peripheral neuropathy, and central nervous system (CNS) events. Some of the adverse events (AEs) associated with lorlatinib have also been reported with other ALK TKIs, including gastrointestinal distress (eg, nausea, vomiting, diarrhea, and constipation) and fatigue (4).

In the phase 3 CROWN study, patients receiving lorlatinib in the first line setting, demonstrated a in a 5-year a progression-free survival (PFS) rate of 60% (95% CI: 51-68) in comparison to 8% (95% CI: 3-14) observed in the crizotinib group. Furthermore, for patients with baseline brain metastasis, lorlatinib demonstrated a higher intracranial response compared with crizotinib (PFS as 53% with lorlatinib and not evaluable with crizotinib as all patients progressed or died or were censored within 2 years). In the lorlatinib group, the probability of being intracranial progression free at 5 years was 83% for patients with baseline brain metastasis and 96% for patients without baseline brain metastasis. Higher incidences of grade 3/4 adverse events (AEs) with lorlatinib than with crizotinib were observed, but permanent discontinuations because of AEs were similar (5).

AEs related to lorlatinib could be primarily driven by its unique chemical structure achieving high intracelluar concentration and very active blood-brain barrier penetration. While the majority of AEs are classified as grade 1–2 (see **Table 1**), effective monitoring and management of these adverse events are paramount to ensuring optimal adherence to lorlatinib treatment, attaining clinical benefit from lorlatinib treatment and mitigating patient risk (1, 6). Most AEs associated with lorlatinib treatment do not require dose interruption or reduction. When necessary, these events are generally managed with temporary dose adjustments. The frequency of such dose reductions varies across clinical trials and real-world settings (7–11).

**Table 1:** Most relevant adverse events related to lorlatinib.

Adverse events	All grades frequency (%)	Grades 3-4 frequency (%)
Metabolic and cardiovascular		
- Hypercholesterolemia	81.1	18.3
- Hypertriglyceridemia	67.2	19.3
- High blood pressure	13.0	6.1
- Hyperglycemia	9.2	3.2
Neurological		
- Peripheral neuropathy	43.7	2.7
- Cognitive impairment	27.7	2.9
- Mood disorders	21.01	1.5
- Speech disorders	8.2	0.6
- Psychotic episodes	6.5	0.4
Gastrointestinal		
- Diarrhea	22.9	1.5
- Nausea	17.6	0.6
- Constipation	17.4	0.2
- Vomiting	12.0	<1
Other		
- Edema	55.7	2.7
- Weight gain	30.9	10.1
- Increased lipase/amylase	12.4/11.3	6.9/2.7
- Fatigue	28.1	0.7
- Pneumonitis	1.9	0.6

A number of guidelines and consensus statements on the management of adverse events caused by lorlatinib have already been published (1, 4, 12, 13). However, none of these focused on Latin America, a region where there are certain variations in terms of the frequency or intensity of toxicities (1).

The objective of this consensus is to provide specific recommendations and pragmatic information for physicians treating these patients in Latin America, adapting international recommendations to the reality of our patients and our institutions. For this reason, both clinical trials and real-world clinical practice experience have been taken into account.

## 2 Methodology

The Delphi method was utilized to develop this document, which was carried out in several phases. First, the available evidence was reviewed based on a literature search in PubMed from January 2010 to July 2025. The search criteria encompassed the term “lorlatinib” in conjunction with “safety,” “adverse events,” “side effects,” “toxicity,” “metabolic alterations,” “dyslipidemia,” “hypertension,” “weight gain,” “neurological,” “central nervous system,” “gastrointestinal,” “edema,” “drug interactions,” “management,” and “guidelines,” along with their respective Spanish equivalents. Priority was given to consensus statements, guidelines, and bibliographic and systematic reviews. The expert group selected references based on their relevance and judiciousness, excluding clinical cases, studies with few patients, and those conducted in specific populations, such as Asian populations.

Subsequently, the coordinators developed a specific questionnaire for this consensus, addressing the criteria that can facilitate the management of adverse events of lorlatinib, taking into account the differential aspects in Latin America that may condition the application of these criteria, in order to issue feasible recommendations to be implemented in the region.

A total of ten oncology specialists were invited to take part in this study. The invitation was extended to them in the form of a survey instrument that was to be submitted electronically. They were selected by each country for being leading experts in the field of lung cancer in their respective countries.

The results were used to draft an initial document for the development of this consensus, which included both the aspects on which agreement was reached in the first round and those on which no agreement was reached, which were left open for further discussion. The document was subsequently returned to the relevant specialists for further review and deliberation on the aspects that had not yet achieved consensus, a subject that had been previously discussed during an online meeting. The decision was made to formulate recommendations only when the level of agreement among the participants reached at least 80% (14, 15). The document was then drafted and approved by all authors.

The results obtained from the aforementioned questionnaire responses are presented in the following segment, accompanied by the recommendations put forth by the expert panel.

## 3 Use of lorlatinib

Lorlatinib has a distinct safety profile from other ALK TKIs, including hyperlipidemia and central nervous system effects. Along with edema and weight gain these are the most common AEs associated with lorlatinib (1, 4). In Latin America, the adverse events most commonly observed in clinical practice are consistent with those reported in the international literature (1, 2, 4, 12, 13). However, there is a scarcity of systematic data regarding the frequency, presentation, or management of these toxicities in real-world practice Considering the clinical characteristics of the Latin American population, which in recent years has exhibited a significant increase in overweight and obesity (almost 75% of adults are overweighted or obese) (16) weight gain associated with lorlatinib can be of significant clinical interest (17–19). Additionally, we can encounter baseline renal alterations in this population. There is no clear recommendation on the dosage of lorlatinib in patients receiving hemodyalisis, but in patients with severe renal insufficiency (estimated glomerular filtration rate of < 30 and not requiring dialysis), the recommended starting dose is of 75mg QD. For patients with mild or moderate renal impairment, no change in the normal recommended starting dose is required (20, 21).

In the context of cancer centers in the region, our observations indicate that the presence of cardiometabolic comorbidities (22) can influence the manifestation and management of certain AEs. This phenomenon is compounded by limitations in timely access to specialized care, including nutrition, mental health, and laboratory services. This consensus aims to initiate a more accurate characterization of these regional differences and offer tools adapted to our real-world setting that optimize treatment adherence and early monitoring of toxicities in patients treated with lorlatinib in Latin America.

It is essential for treating physicians to be familiar with the types of AEs that are likely to occur within specific timeframes. Furthermore, it is important to inform and prepare patients and care partners about what to expect while taking lorlatinib. This includes the type and likelihood of AEs and the frequency of evaluations (3, 4).

Due to the broad spectrum of lorlatinib-related AEs, appropriate management requires the involvement of a multidisciplinary group of specialists.The main recommendation for the management of each of the AEs associated with lorlatinib is to follow the guidelines of the summary of product characteristics (SPC). However, there are situations in which it is necessary to approach the management of AEs individually, to avoid possible drug interactions with concomitant treatments or to preserve the patient’s quality of life, which can be greatly impaired, especially by neurocognitive AEs (1, 23).

Subsequent to the initiation of treatment with lorlatinib, patients should undergo monitoring at each follow-up visit for AEs commonly associated with lorlatinib. From a practical perspective, the evaluation of AEs should concentrate on the risk they pose to the patient, the extent to which the AE impacts the patient, and the level of functional impairment it causes. The perceived impact on the patient is subjective, depending on their baseline functioning, daily habits, and usual activities. In contrast, functional impairment can be assessed more objectively by evaluating specific abilities (e.g., difficulties with speech, cognition, or performing tasks like buttoning a shirt). The more severe the symptoms and functional limitations, the more likely it is that intervention strategies or dose adjustments will be necessary. Once AEs are identified during monitoring, the next step is to manage them appropriately (4, 13).

In clinical trials, most lorlatinib AEs, including laboratory abnormalities, were effectively managed or reversed with mitigation strategies or dose modifications. Mitigation strategies, encompassing lifestyle modifications or pharmacological interventions, may be implemented concurrently. When mitigation strategies are not effective in reversing the AE or lowering it to grade 1 or 2, it may be necessary dose modification, which includes dose reductions and/or interruptions. Grade 3 and 4 can be more difficult to manage compared to managing grade 1 and 2 AEs, requiring more frequent dose modifications. Follow-up frequency may vary, generally occurring more frequently during the window of onset of grade 3 AEs. The monitoring process should prioritize not only the identification of AEs but also the assessment of their severity. Given the potential for neuropsychiatric AEs, the provision of social support and the assurance of companion presence during evaluations are of paramount importance (4).

Temporary interruptionof lorlatinib is generally preferable to reducing the dosage and waiting for toxicity to improve before restarting. In the case of non-laboratory-related AEs (mainly CNS AEs) requiring dose reductions, mitigation strategies should be continued if symptoms persist as moderate or severe, and the dose may be further reduced if they persist. If symptoms improve, the lorlatinib dosage should be maintained or, in accordance with the healthcare professional’s clinical assessment, increased if the mitigation strategies appear to be effective or if disease control is compromised (4).

For non-laboratory-related AEs requiring dose interruption, the temporary interruption of the dosage should persist until the symptoms of the AE subside to the extent that the patient experiences only mild discomfort or is functionally unaffected (or has no discomfort or has regained function). This process generally requires several weeks to complete. During the interruption of the dosage, toxicity improvement should be assessed approximately twice weekly (4).

### 3.1 Recommendations

- Before starting treatment with lorlatinib, it is important that patient understands the potential AEs.
- Before starting treatment, a neurological evaluation and brain magnetic resonance imaging (MRI) must be performed, as well as a cardiovascular function assessment via electrocardiogram. Blood pressure must be measured as well as serum cholesterol and triglyceride levels. Comorbidities and concurrent medications must also be assessed.
- If AEs arise during monitoring and the symptoms are not bothersome or are only mild, mitigation strategies are preferred over reducing the dose or temporarily interrupting treatment.
- When a dose reduction is appropriate, the initial lorlatinib dose of 100 mg per day may be reduced to 75 mg per day, followed by a further reduction to 50 mg per day in case needed.
- Patients who experience AEs requiring dose modification should be re-evaluated at least once a month until symptoms improve.
- Once the AEs has resolved, the possibility of restarting at the same or a lower dose level is evaluated, depending on the degree and type of toxicity.

## 4 Hyperlipidemia

Hyperlipidemia has been identified as the most common AE associated with lorlatinib, with a median onset time of 15 days and a duration of more than 400 days (1, 4). In the CROWN study, increased blood lipid levels were reported as high cholesterol in 72% of patients and as high triglycerides in 66% of patients (6).

Although dietary modifications may be employed, hyperlipidemia typically requires pharmacological interventions. In the presence of mild or moderate hypercholesterolemia or mild or moderate hypertriglyceridemia, lipid-lowering treatment should be initiated (or modified if the patient is already receiving treatment). In cases of severe hypercholesterolemia or severe hypertriglyceridemia, lipid-lowering treatment should be initiated; if the patient is already receiving lipid-lowering treatment, increase the dose of this treatment according to the corresponding SPC, or switch to a new lipid-lowering treatment. The same dose of lorlatinib should be continued without interruption. Only in cases of life-threatening hypercholesterolemia or hypertriglyceridemia, lorlatinib treatment should be interrupted until hypercholesterolemia and/or hypertriglyceridemia reach moderate or mild severity, and then treatment should be restarted at the same dose of lorlatinib while maximizing lipid-lowering therapy according to the corresponding SPC (20).

The preferred treatment option is a statin (1, 3). We start with a statin, usually rosuvastatin which is available thoughout the region. In the event that monotherapy with a statin at the maximum tolerated dose proves inadequate in controlling cholesterol or triglyceride levels, the addition of a second drug may be indicated, contingent upon the patient’s profile. In such cases, ezetimibe or fibrates may be considered as appropriate (1, 4).

In the event that a patient exhibits intolerance to statins or does not demonstrate a satisfactory response to maximal lipid-lowering therapeutic interventions, referral to a specialized cardiovascular or lipid clinic is recommended. In such cases, medical specialists may prescribe PCSK9 inhibitors or other alternative lipid-lowering therapies (4).

Following management in the CROWN study (6), most hyperlipidemia events (73%) improved or were completely resolved (lipid levels were brought back to normal levels with management strategies). Additionally, the high rate of hyperlipidemia has not been associated with an increased likelihood of side effects affecting the heart or blood vessels (cardiovascular) so far (13).

### 4.1 Recommendations

- Lipid levels should be measured before treatment begins, two weeks after treatment initiation, and monthly thereafter.
- Patients with hyperlipidemia should be treated with rosuvastatin as a first-line therapy (pitavastatin and pravastatin are not available in many Latinoamerican countries). In patients with moderate renal impairment (creatinine clearance <60 ml/min), the recommended starting dose is 5 mg. The 40 mg dose is contraindicated in patients with moderate renal impairment. In patients with severe renal impairment, the use of rosuvastatin is contraindicated at any dose. Although rosuvastatin is not contraindicated in patients on hemodialysis, it is recommended to start at low doses (5 mg/day). Statins that interact more with cytochrome P450 should be avoided.
- In patients with persistent hypercholesterolemia despite statin treatment, ezetimibe should be added.
- In patients with triglyceride levels of at least 500 mg/dL, add fibrates to statin treatment.
- In patients intolerant to statins, re-exposure with a different statin at a low or intermittent dose should be attempted. PCSK9 inhibitors should be considered in select cases.
- Temporary interruption of lorlatinib therapy should be considered for patients with severe hyperlipidemia that remains uncontrolled despite optimized medical treatment.
- Dose reduction of lorlatinib due to dyslipidemia should only be considered if severe hyperlipidemia recurs despite optimized medical treatment.

## 5 Weight gain

Mild to moderate weight gain, ranging from 10% to 20%, is another common AE associated with lorlatinib (1). This effect is typically observed within the first two months after the start of treatment (3). In the CROWN trial, weight gain frequency was 38% (7% grade 1, 14% grade 2, 17% grade 3) at 18.3 months, increasing to 44% (21% grade 1–2, 23% grade 3) reported at 5 years follow-up. Grade 3 weight gain was defined as a ≥20% increase from baseline, during treatment with lorlatinib (6).

Weight gain has the potential to substantially impact a patient’s quality of life. Alongside increased risk of mortality, obesity is associated with the development of serious comorbidities including cardiometabolic (type 2 diabetes mellitus [T2DM], hypertension, cardiovascular disease), biomechanical (obstructive sleep apnea, knee and hip osteoarthritis), and psychosocial conditions (24).

In certain instances, weight gain and edema manifest concurrently. In such cases, the treatment of edema should be prioritized, as its reduction may subsequently impact body weight (1, 4).

The management of weight gain is achieved through non-pharmacological approaches, such as dietary modifications and regular exercise (1, 12). In the context of weight gain, there is an absence of a particular medical intervention that is specifically designed to address this issue. In general, there is no necessity to interrupt or reduce the dosage of lorlatinib (1).

### 5.1 Recommendations

- In patients treated with lorlatinib who experience weight gain, it is advisable to implement non-pharmacological approaches, including personalized nutritional intervention, regular physical activity, and education on healthy habits.
- In patients with secondary hyperglycemia and a history of T2DM or cardiovascular disease, iSGLT2s (empagliflozin, canagliflozin, or dapagliflozin) and GLP-1 receptor agonists (GLP-1 RAs) (liraglutide or semaglutide) may be used. However, referral to an endocrinologist for an expert assessment is advisable.
- The utilization of semaglutide or other GLP-1 agonists (incretins) may be contemplated in patients undergoing treatment with lorlatinib who have experienced substantial weight gain or metabolic syndrome. Nevertheless, it is advised that a referral to an endocrinologist for an expert assessment be considered.
- The discontinuation or reduction of lorlatinib is only indicated in cases of severe illness or cases that are unresponsive to conservative management, particularly when there is a substantial functional or metabolic impact.
- In cases of progressive and clinically significant weight gain (e.g. >20%), especially if associated with functional impairment or comorbidities, temporary interruptionof lorlatinib may be considered, along with the implementation of specific medical and nutritional measures. The determination of the most appropriate course of action should be informed by a throughout clinical evaluation.

## 6 Hypertension

Hypertension can develop for anyone taking lorlatinib and can get worse for patients with pre-existing hypertension or cardiovascular disease, advanced age, a history of smoking, hyperlipidemia, and/or obesity (1). In the CROWN trial, any grade hypertension occurred in 26% of patients, being grade 3 in 12% (6).

According to cardio-oncology guidelines, it is imperative that all cancer patients maintain blood pressure levels < 140/90 mmHg (or < 130/80 mmHg in cases of very high cardiovascular risk) (25).

In patients receiving treated with lorlatinib, blood pressure should be monitorised 2 weeks after initiation and at least once a month thoughout (1). In grade 3 hypertension (≥160/100 mmHg) with more than one antihypertensive agent or more intensive treatment than the previous one, lorlatinib should be temporarily interrumped until the hypertension has resolved to grade 1 or lower. If grade 3 hypertension recurs, lorlatinib treatment should be temporarily interrumped until it resolves to grade 1 or lower, and then treatment should be resumed at a reduced dose. If adequate control of hypertension cannot be achieved with optimal medical therapy, lorlatinib treatment should be permanently interrumped. In grade 4 hypertension (life-threatening consequences, urgent intervention indicated), if hypertension recurs after temporary interruption or dose reduction, lorlatinib should be permanently interrumped (20).

The initial treatment approach for hypertension management in cancer patients should entail the prescription of an angiotensin-converting enzyme inhibitor (ACEI) or an angiotensin II receptor blocker (ARB), as these pharmaceutical agents have been demonstrated to exhibit a more favorable profile in terms of reducing the risk of diabetic nephropathy or ventricular dysfunction (25).

### 6.1 Recommendations

- It is imperative that blood pressure levels in patients treated with lorlatinib be individualized, taking into account their comorbidities and adhering to international guidelines. As a general recommendation, blood pressure should be maintained below140/90 mmHg in patients without intercurrent cardiovascular disease and below 130/80 mmHg in those with other conditions (chronic kidney disease, T2DM, among others).
- Blood pressure should be monitored at each visit during the first month of lorlatinib treatment, especially for patients with a history of or high risk for cardiovascular disease. Depending on clinical stability, monitoring frequency may be reduced (e.g., monthly), though active monitoring is recommended throughout treatment in all cases.
- For grade 2 hypertension (<160–100 mmHg), initiate treatment with an ACEI or ARB, then continue with lorlatinib. If adequate control is not achieved, add a calcium channel blocker.
- In patients with grade 3 hypertension, initiate or intensify antihypertensive treatment while continuing lorlatinib with close clinical monitoring. Only temporary interruption is recommended if adequate control is not achieved or symptoms appear.
- If blood pressure control is not achieved with antihypertensive medication, as with any AE, the dose of lorlainib could be reduced to seek blood pressure control.
- In grade 4 hypertension, lorlatinib should be temporarily interrumped until complete clinical stabilization is achieved.

## 7 Edema

Edema is one of the most common AEs in patients receiving lorlatinib. In the CROWN study, edema occurred in more than half of the patients taking lorlatinib (57%), with symptoms first appearing on average around 1.8 months on treatment (6). Most cases were mild or moderate, with only 4% of patients reporting severe edema (1, 3, 6). Peripheral edema is the most common presentation of the condition; however, cases of facial and periorbital edema have also been reported (3).

In practice, determining how much edema affects the patient or their daily functioning is a more useful approach than establishing the degree of severity. Patients should be regularly evaluated for conditions that may lead to edema, including cardiac, renal, or thyroid issues. If edema is detected but not bothersome, lifestyle changes may be sufficient. For mildly to moderately bothersome edema, therapeutic options like physiotherapy, lymphatic massage, or compression stockings are recommended. Although evidence for diuretic use is limited, they may be considered based on clinical experience (4).

Patients with symptoms of bothersome edema should be reassessed approximately monthly and mitigation strategies modulated based on symptom severity. In patients with severely bothersome edema that has not improved upon reassessment, the lorlatinib dose should be further reduced in 25-mg decrements. If symptoms of severe edema improve, lorlatinib therapy can be maintained or, based on clinical judgement, possibly increased if the dose is ≤75 mg, with routine monitoring (4).

### 7.1 Recommendations

- Once treatment with lorlatinib has begun, it is recommended that edema be monitored at each visit to ensure that it is not caused by other factors.

- There is no clear evidence for the management of grade 1–2 edema, so only physical measures, such as compression stockings or elevating the feet while resting, can be recommended.

- For edema grade <3, the following are recommended:

o Non-pharmacological measures, such as compression garments, elevation of the affected area above heart level, increased exercise, sodium reduction, physical therapy, and massage for lymphedema.
o In the presence of lorlatinib-associated edema, start treatment with diuretics if there are functional symptoms, and continue treatment with lorlatinib if the edema is grade 1–2.
o NT-proBNP measurement may be considered in patients with clinical signs of cardiovascular involvement.

- In grade ≥ 3 edema, the following are recommended:

o NT-proBNP assessment.
o Echocardiogram.
o Temporal interruption of lorlatinib until symptoms reach grade ≤ 2.

## 8 Peripheral neuropathy

Peripheral neuropathy associated with lorlatinib was observed in around 40% of patients in the CROWN study, manifesting as mild symptoms that are typically reversible upon modification of the lorlatinib dosage. On average, symptoms of peripheral neuropathy began around 3.7 months. The condition manifests primarily as paresthesia, although patients may also experience peripheral sensory neuropathy and muscle weakness. The symptoms are frequently characterized as tingling, numbness, and nocturnal pain. These symptoms typically appear initially in the fingers and toes and subsequently extend proximally to the extremities (3, 4, 12).

Prior to the start of treatment, patients should undergo evaluation for any underlying causes of peripheral neuropathy, such as diabetes. At each follow-up visit, patients should be re-evaluated to ascertain the presence of signs and symptoms indicative of peripheral neuropathy (4).

The management of peripheral neuropathy is mainly based on its severity. For grade 1 and 2 AEs, reducing the dose of lorlatinib is the best choice. For grade ≥3 peripheral neuropathy, treatment should be temporarily interrumped and enforced until reduced to grade ≤2 or returned to baseline. For patients with severe symptoms, some medical therapy is needed. Vitamin B1 and vitamin B6 and medications for pain associated with peripheral neuropathy are useful for relieving symptoms (26).

### 8.1 Recommendations

- There is no evidence recommending non-pharmacological treatments; however, rehabilitation and occupational therapy, physical therapy, warming of the extremities, and nutritional measures may alleviate symptoms.
- In grades 1 and 2 neuropathy, lorlatinib should be continued without dose modification.
- In grade 3 or higher neuropathy, temporarily interruptlorlatinib until symptoms resolve to grade 2 or lower.
- To relieve symptoms, medications for pain associated with peripheral neuropathy, such as gabapentin, pregabalin, or duloxetine, may be used.
- Also, to relieve symptoms, vitamin B1 and B6 are recommended in addition to the aforementioned measures.
- Edema should be ruled out as a cause of compression neuropathy, and if present, it should be treated.
- If edema is present, duloxetine is the drug of choice.
- If edema is present, follow the recommendations for edema management.

## 9 Effects on the central nervous system

While the high penetration of lorlatinib into the central nervous system (CNS) provides a treatment advantage, it also provides a basis for CNS AEs. Furthermore, brain metastases, which are frequently observed in patients diagnosed with ALK-positive non-small cell lung cancer, have been demonstrated to potentially contribute to the manifestation of neurological symptoms and signs. Therefore, it is advisable, when available, to conduct a comprehensive neurological evaluation and obtain a brain MRI prior to the initiation of treatment with lorlatinib in order to rule out brain metastasis (23, 27, 28).

In the CROWN study, all-causality CNS AEs occurred in 42% of patients in the lorlatinib group, the majority of which (86%) were of grade 1/2 severity. Only three patients who experienced treatment-related CNS AEs (two had confusional state and one had nightmares) permanently interrupted lorlatinib. In the lorlatinib group, CNS AEs occurred in six of nine (67%) patients who had prior brain radiotherapy and in 57 of 140 (41%) patients without prior brain radiotherapy (6).

In many cases, the effects on the CNS resolve without intervention, with concomitant medication, dose modification, or a combination of both (5, 12, 23). According to the SPC, for grade 2 or 3 AEs, lorlatinib treatment should be interrupted until toxicity is ≤ grade 1. Afterward, lorlatinib treatment can be resumed at a reduced dose. For grade 4 toxicity, lorlatinib should be permanently interrumped (20) In all studies conducted with lorlatinib, a reduction in dosage has been demonstrated to mitigate the occurrence of most neurocognitive AEs without compromising the medication’s effectiveness (23, 29).

From a patient perspective, it is important to discuss the individual categories of CNS side effects based on their presentation, which will help patients and care partners to recognize them (13).

A rapid diagnosis of CNS AEs, is crucial, as early temporary treatment interruption can lead to rapid reversal of such AEs, especially in combination with a dose reduction at the time of reinitiation of lorlatinib therapy (4).

### 9.1 Recommendations

- It is recommended that patients be accompanied for the assessment of neuropsychiatric toxicities.
- When evaluating patients, it is important to identify factors that may increase the risk of neurological toxicity, such as brain metastases, radiotherapy, brain surgery, psychiatric medications, and opioids.
- The patient and their family should be informed about how to identify adverse neurological reactions.
- An evaluation by a psychiatrist or neurologist should be recommended depending on toxicity control and each patient’s personal situation.
- In cases of grade 1 toxicity, treatment with lorlatinib should continue at the current dose. If this is not possible, a lower dose should be considered depending on the patient’s profile.
- In the presence of grade 2–3 toxicity, treatment should be temporarily interrupted until symptoms resolve to grade ≤2. Once controlled, reintroduction with a reduced dose may be considered, with close neurological monitoring.
- In cases of grade 4, permanently interrupting lorlatinib may be necessary.

#### 9.1.1 Cognitive effects

Cognitive impairments associated with lorlatinib include memory impairment, mental impairment, confusion, amnesia, attention disorders, delirium, and dementia (1, 27, 28).

In the CROWN study, cognitive effects occurred in 28% of patients in the CROWN study, with mild or moderate presentation in most patients (6); the average time to onset was 5.1 months. Most CNS effects (56%) were resolved with either no intervention, concomitant medication, dose modification, or a combination (4).

##### 9.1.1.1 Recommendations

- In patients with grade 1 symptoms, the dose can be maintained with close monitoring since some patients improve over time.
- In cases of grade 2 or 3 toxicity, treatment should be temporarily interrupted. Once symptoms improve, lorlatinib can be restarted at a reduced dose.
- In cases of grade 4, permanently interrupting lorlatinib may be necessary.
- In some cases, vortioxetine or duloxetine may be indicated.

#### 9.1.2 Mood disorders

SMood disorders include affective disorders, affective instability, aggression, nervousness, irritability, anxiety, bipolar disorder type I, depressed mood, depression, euphoria, mania, mood disturbance, mood swings, personality changes, and stress. In the CROWN study, mood disorders occur in 16% of patients treated with lorlatinib, with the majority being grade 1–2. Median time to onset was 1.2 months. Most of the effectsare reversible (1, 3, 6, 28).

##### 9.1.2.1 Recommendations

- In the presence of grade 1 toxicity, it is recommended that the lorlatinib dose be maintained or reduced.
- In the presence of grade 2 to 3 toxicity, treatment with lorlatinib should be interrumped until the toxicity is grade 1 or lower, at which point treatment can resume at a lower dose.
- If toxicity is grade 4, treatment with lorlatinib should be permanently interrumped.
- For patients with mild anxiety, non-pharmacological measures such as relaxation techniques, cognitive behavioral therapy, or psychological support may be sufficient. For moderate or persistent anxiety, short-acting anxiolytics (such as lorazepam) or antidepressants with anxiolytic effects (such as sertraline or escitalopram) may be recommended.
- In patients with mild depression, non-pharmacological measures such as psychological support, cognitive behavioral therapy, and close monitoring by the healthcare team may be initiated. If depression is moderate or severe, treatment with antidepressants tailored to the patient’s profile is recommended, as well as referral to a psychiatrist for an expert assessment.

#### 9.1.3 Speech disorders

The effects of lorlatinib on speech include dysarthria, slow speech, and impaired speech (difficulty finding words). In the CROWN study, speech disorders occurred in 6% of patients, with mild to moderate presentation in all but 1 patient (1%) (6); the average time to onset was 3.5 months (4). The management of these disorders is contingent upon the patient’s ability to communicate effectively and the nature of the disorder’s presentation, whether it is continuous or intermittent (1).

##### 9.1.3.1 Recommendations

- In grade 1 toxicity, close observation and monitoring are recommended, and treatment with lorlatinib should continue.
- In grades 2-3, treatment with lorlatinib should be temporarily interrumped until the toxicity is grade 1 or lower, at which point treatment can resume at a lower dose.
- In grade 4 toxicity, lorlatinib should be permanently interrumped.
- A non-drug-related neurological cause should always be ruled out, and support from a neurologist or speech therapist may be helpful.

#### 9.1.4 Psychotic episodes

The most common psychotic episodes associated with lorlatinib are hallucinations, which can present as an individual hearing, smelling, or seeing something that is not actually there (13), and most cases are grade 1–2 (23). In the CROWN study, psychotic episodes occurred in 5% of patients, with most patients reporting mild or moderate symptoms. The average time to onset was 1.4 months (6).

##### 9.1.4.1 Recommendations

- In grade 1 toxicity, treatment with lorlatinib should continue.
- In grades 2 and 3, it is recommended to temporarily interrupt lorlatinib, seek a psychiatric evaluation, and administer antipsychotics (such as olanzapine).
- In grade 4 toxicity, permanent interruption of lorlatinib and an urgent psychiatric evaluation are recommended.

## 10 Increased amylase levels

Increases in amylase and/or lipase levels have been detected in approximately 12% of patients receiving lorlatinib, most of which were grade ≤2 (1). The median time to onset of increased lipase and serum amylase levels is 141 days and 138 days, respectively (20).

Levels of amylase and/or lipase should be monitorised prior to initiation of lorlatinib and thereafter on a regular basis. Only when toxicity is grade 3 should lorlatinib be temporarily interrupted until symptoms resolve to less than or equal to grade 2 or baseline, and then treatment should be resumed at a reduced dose. If the patient is asymptomatic and does not have pancreatitis, treatment modification will depend on the physician’s judgment (1).

### 10.1 Recommendations

- Monitoring amylase and lipase levels is not routinely performed for all patients treated with lorlatinib, but it should be considered in specific clinical situations due to the risk of pancreatitis, even if low, induced by severe hypertriglyceridemia, a known adverse effect of this drug.
- Continuing treatment with lorlatinib is indicated in most cases, even with grade 1–2 adverse effects, unless there are clinical symptoms.
- In cases of grade 3 or 4 toxicity, or in the presence of clinical symptoms suggestive of pancreatitis, treatment with lorlatinib should be interrumped.

## 11 Gastrointestinal effects

It has been reported that between 10% and 20% of patients experience gastrointestinal AEs (e.g., diarrhea, nausea, constipation, and vomiting) when undergoing treatment with lorlatinib. The EAs are generally mild in nature and can be managed through the implementation of general measures. Such measures may include dietary adjustments and standard medical therapy (1, 3).

### 11.1 Recommendations

- For grade <3 toxicity, it is recommended that treatment with lorlatinib continue.
- In cases of grade ≥3 toxicity, the dose should be interrupted until symptoms resolve to grade ≤2, at which point it can be restarted at a reduced dose.
- In cases of diarrhea, loperamide and a low-fiber, low-fat, low-irritant diet are recommended, in addition to ensuring adequate oral hydration. If diarrhea is mild or moderate (grades 1–2), this management is usually sufficient, and the lorlatinib dose can be maintained. In cases of persistent diarrhea or grade ≥3 diarrhea, treatment should bei temporarily interrupted, infectious causes should be ruled out, and more intensive management should be initiated. Reintroduction of lorlatinib at a reduced dose should be considered once symptoms are under control.
- In cases of constipation, non-pharmacological measures such as increased fluid intake, a high-fiber diet, and regular physical activity are recommended if the patient’s condition permits. If these measures are insufficient, mild laxatives such as polyethylene glycol or lactulose may be indicated depending on tolerance and response.
- For managing nausea and vomiting, antiemetics such as ondansetron, metoclopramide, or domperidone are recommended initially by the oral or sublingual route. Parenteral administration is recommended if symptoms persist.

## 12 Hyperglycemia

The prevalence of hyperglycemia resulting from treatment with lorlatinib is 9%. Patients exhibiting the highest degree of risk are those with a documented medical history of diabetes, coronary artery disease, heart disease, or obesity (1). Hyperglycemia is usually managed with lifestyle changes, a diabetes-appropriate diet, and, in grade 2, oral antidiabetic agents. Only in grade 3 and 4 hyperglycemia should treatment be temporarily interrumped until hyperglycemia is adequately controlled, and then lorlatinib should be resumed at the next lowest dose. If adequate hyperglycemic control cannot be achieved with optimal medical therapy, lorlatinib should be permanently interrumped (20).

### 12.1 Recommendations

- HbA1c levels should be evaluated prior to the initiation of treatment with lorlatinib and subsequently at three- to six-month intervals.
- A preliminary evaluation of blood glucose levels may be conducted, a procedure that is both cost-effective and conducive to enhanced interpretation of subsequent abnormalities in follow-up studies.
- In addition to modifications in lifestyle, metformin is a pharmacological agent that deserves consideration as a first-line treatment for patients who do not have a history of diabetes and who exhibit only mild hyperglycemia. This recommendation is due to the medication’s efficacy, its low risk of hypoglycemia, and its favorable metabolic profile. In more severe cases or in patients with known diabetes, it may be necessary to intensify treatment with GLP-1 agonists, SGLT2 inhibitors, or even insulin if glucose levels are very high or symptomatic. In such cases, referral to a specialist is recommended.
- If diabetes is diagnosed, it is recommended that the patient be referred to an endocrinologist.
- In instances of severe toxicities (grade ≥3) that fail to respond to medical management and compromise the patient’s safety or quality of life, the temporary interruption of lorlatinib should be considered.
- Following the temporary interruption of lorlatinib treatment, once glycemic control has been achieved, medication intake should be restarted at a reduced dosage.

## 13 Fatigue

Fatigue occurs in approximately 20% of patients treated with lorlatinib (4). Only if fatigue is severe, lorlatinib treatment should be temporarily interrupted until symptoms resolve to ≤ grade 2 or baseline, and then lorlatinib treatment should be resumed at a reduced dose level (20).

### 13.1 Recommendations

- In patients presenting with fatigue, a recommended course of action includes a routine of light to moderate physical activity, alongside the promotion of optimal sleep hygiene practices, the division of daily activities to avoid overload, the assurance of adequate nutrition, and the treatment of reversible causes, such as anemia, hypothyroidism, and hypercalcemia.
- Temporary interruption of lorlatinib should only be considered in the presence of grade 3–4 toxicity.

## 14 Interstitial lung disease/pneumonitis

Interstitial lung disease, lung opacity, and pneumonitis have been observed in a small percentage of patients treated with lorlatinib. To date, no radiological or temporal pattern of pneumonitis has been described for lorlatinib (1). The prevalence of interstitial lung disease/pneumonitis in patients who have received lorlatinib is documented as 1.9%. In grades 1 and 2, temporary interruption of treatment and subsequent reinitiation at a reduced dose with steroids may be considered. Treatment with lorlatinib should be permanently interrumped for any grade of interstitial lung disease/pneumonitis if it recurs or does not improve after 6 weeks of treatment with lorlatinib and steroids (20).

### 14.1 Recommendations

- Physicians should check for a history of lung disease, previous radiation therapy, or immunotherapy in patients who develop pneumonitis, as these could explain the development of pneumonitis.
- If pneumonitis is caused by prior treatment, the full lorlatinib dose may be used.
- Lorlatinib should be discontinued if pneumonitis is confirmed as an adverse event of lorlatinib and is grade 3 or higher.
- In grade 3 AEs that have been treated with immunotherapy and respond to medical management, the reintroduction of lorlatinib should be evaluated on a case-by-case basis, especially in patients who have a clinical response and CNS involvement.
- To monitor pneumonitis, perform clinical and radiological follow-up one week after restarting lorlatinib treatment until pulmonary toxicity resolves.

## 15 Drug interactions

Lorlatinib is metabolized in the liver, specifically by cytochrome P450 (CYP) 3A4, uridine diphosphate-glucuronosyltransferase (UGT) 1A4, and to a lesser extent by CYP2C8, CYP2C19, CYP3A5, and UGT1A3. It is crucial to consider this aspect when administering treatments that involve the same pathway or interfere with inducers or inhibitors of the same pathway, as this could impact the plasma concentrations of lorlatinib (1, 26, 30). The primary P450 3A inducers and inhibitors are shown on **Table 2** (31).

**Table 2:** Cytochrome P450 3A inhibitors and inducers (including 3A4).

Potent inducers	Moderate inducers	Potent inhibitors	Moderate inhibitors
Apalutamide Carbamazepine Encorafenib Enzalutamide Phenytoin Fosphenytoin Lumacaftor Lumacaftor-ivacaftor Mitotane Rifampin	Bexarotene Bosentan Cenobamate Dabrafenib Dexamethasone Dipyrone Efavirenz Elagolix, estradiol, and norethindrone Eslicarbazepine Etravirine Phenobarbital Fexinidazole Lorlatinib Mitapivat Modafinil Nafcillin Pacritinib Pexidartinib Primidone Repotrectinib Rifabutin Rifapentine Sotorasib St. John’s wort	Adagrasib Atazanavir Ceritinib Clarithromycin Cobicistat Darunavir Idelalisib Indinavir Itraconazole Ketoconazole Levoketoconazole Lonafarnib Lopinavir Mifepristone Nefazodone Nelfinavir Nirmatrelvir-ritonavir Ombitasvir-paritaprevir-ritonavir Ombitasvir-paritaprevir-ritonavir + dasabuvir Posaconazole Ritonavir Saquinavir Tucatinib Voriconazole	Amiodarone Aprepitant Avacopan Berotralstat Cimetidine Conivaptan Crizotinib Cyclosporine Diltiazem Duvelisib Dronedarone Erythromycin Fedratinib Fluconazole Fosamprenavir Fosaprepitant Fosnetupitant-palonosetron Imatinib Isavuconazole Lefamulin Letermovir Netupitant Nilotinib Nirogecestat Ribociclib Schisandra Verapamil Grapefruit juice

### 15.1 Recommendations

- Concomitant use with potent CYP3A4/5 inducers should be avoided because they may reduce lorlatinib’s plasma concentration, thereby decreasing its efficacy.
- Concomitant use of CYP3A4/5 inhibitors should also be avoided because they may increase lorlatinib concentrations.
- Concomitant administration of lorlatinib with CYP2B6 substrates, such as bupropion and efavirenz, should be avoided, as it may result in reduced plasma concentrations of the CYP2B6 substrate.
- Drugs metabolized by CYP2C9 and UGT with a narrow therapeutic index, such as coumarin and anticoagulants, should be used with caution.
- Drugs that are P-glycoprotein substrates with a narrow therapeutic index, such as digoxin and dabigatran, should also be used with caution.
- Exposure to lorlatinib is not significantly altered by the presence of mild liver damage or mild to moderate kidney failure, so no dose adjustments are recommended for patients with these conditions.
